# DNA repair deregulation in discrete prostate cancer lesions identified on multi-parametric MRI and targeted by MRI/ultrasound fusion-guided biopsy

**DOI:** 10.18632/oncotarget.19145

**Published:** 2017-07-10

**Authors:** Caleb R. Dulaney, Soroush Rais-Bahrami, Debra Della Manna, Jennifer B. Gordetsky, Jeffrey W. Nix, Eddy S. Yang

**Affiliations:** ^1^ Department of Radiation Oncology, University of Alabama at Birmingham, Birmingham, AL, USA; ^2^ Department of Urology, University of Alabama at Birmingham, Birmingham, AL, USA; ^3^ Department of Radiology, University of Alabama at Birmingham, Birmingham, AL, USA; ^4^ Department of Pathology, University of Alabama at Birmingham, Birmingham, AL, USA; ^5^ Department of Cell, Developmental, and Integrative Biology, University of Alabama at Birmingham, Birmingham, AL, USA; ^6^ Department of Pharmacology and Toxicology University of Alabama at Birmingham, Birmingham, AL, USA

**Keywords:** prostatic neoplasms, DNA repair, magnetic resonance imaging, image-guided biopsy

## Abstract

Prostate cancer is histologically and molecularly heterogeneous. Clinically significant disease is often driven by dominant intra-prostatic lesions (IPLs). Prostate cancers cluster into molecular phenotypes with substantial genetic heterogeneity making pathway-based molecular analysis appealing. MRI/ultrasound fusion biopsy provides a unique opportunity to characterize tumor biology of discrete lesions at diagnosis. This study determined the feasibility of pathway-based gene expression analysis of prostate biopsies and characterized cancer pathway deregulation.

Thirteen patients had prostate cancer diagnosed by MRI/ultrasound fusion biopsy and either Gleason 6 or Gleason ≥8. Gene expression profiling was performed on 14 biopsies using >700 genes representing 13 cancer pathways. Pathway-based analysis compared gene expression among samples based on clinical, pathological, and radiographic characteristics. Pathway-based gene expression analysis was successful in 12 of 14 (86%) samples. Samples clustered based upon deregulation of DNA Repair and Notch, Chromatin Modification and Cell Cycle, or all other pathways, respectively. DNA Repair demonstrated the greatest differential deregulation. Lesions with Gleason ≥8, PSA ≥10, or intense dynamic contrast enhancement (DCE) had significantly higher DNA Repair deregulation than those with Gleason 6, PSA <10, or low to moderate DCE. Alterations in DNA Repair gene expression were diverse with upregulation of markers of DNA damage and down-regulation of DNA Repair proteins. This study demonstrates the feasibility of pathway-level gene expression analysis of discrete intra-prostatic lesions sampled by MRI/ultrasound fusion biopsy. IPLs cluster into distinct molecular phenotypes, the most significantly altered being DNA Repair.

## INTRODUCTION

Despite decades of advancement in prostate cancer management, distinguishing men with aggressive, clinically-significant disease at the time of diagnosis remains challenging. Many men with low-risk, indolent prostate cancer have historically been over-treated. Even in the modern era of risk stratification and advanced treatment options, men with high-risk disease continue to develop recurrence, symptomatic progression, or die of their disease. Diagnosis of prostate cancer classically relies on digital rectal examination (DRE), serum prostate-specific antigen (PSA) levels, and pathologic evaluation of standard biopsy samples. The current management approach relies very little on understanding tumor biology and there is significant heterogeneity in disease behavior within current risk stratification groups [1–3]. Personalizing therapy by understanding tumor biology could better guide management and aid in shared decision-making, including discussions of active surveillance and primary definitive therapy options in appropriate cases.

The standard extended-sextant prostate biopsy technique is essentially a systematic, but otherwise random sampling of various regions of the prostate gland. Prostate cancer is an extremely heterogeneous spectrum of disease, and the majority of men with prostate cancer harbor multiple foci of disease [4–6]. However, the natural course of most clinically significant prostate cancer appears to be driven by dominant intra-prostatic lesions (IPL) [4–6]. Advances in multi-parametric prostate MRI (mpMRI) have allowed for identification and radiographic risk stratification of IPLs [7–10]. Real-time fusion of mpMRI with trans-rectal ultrasound (TRUS) now allows urologists to perform targeted biopsies of IPLs [11]. The targeted biopsy technique improves detection of clinically significant foci of prostate cancer over the systematic biopsy schema, even in cases of otherwise occult disease [12, 13].

Emerging data supports the clustering of large proportions of prostate cancer into molecular phenotypes, similar to the well-known molecular phenotypes used to describe breast cancer [14]. Many emerging cancer therapies target specific cellular pathways that drive cancer progression. In fact, molecular stratification of metastatic prostate cancer provides important information that can be used to tailor targeted treatment approaches [15]. Cancer, in general, is driven by an accumulation of genetic mutations that modulate important molecular pathways promoting tumorigenesis, termed canonical cancer pathways [16]. While many archetype genes drive pathway deregulation in various malignancies, prostate cancer is characterized by substantial molecular heterogeneity making cancer pathway analysis, as opposed to individual gene analysis, more appealing [14]. The purpose of this study was to characterize and compare canonical cancer pathway deregulation using the Nanostring platform in malignant IPLs sampled by targeted MRI/TRUS fusion biopsy with the hypothesis that pathologically proven high-grade IPLs would have greater pathway deregulation than low-grade IPLs. This study also explored clinical and radiographic correlates of tumor biology and cancer pathway deregulation.

## RESULTS

### Clinical and radiographic characteristics

Thirteen patients with fourteen discrete IPLs were identified who had undergone targeted MRI/TRUS fusion biopsy and were found to have GS (Gleason score) 6 (7 IPLs) or GS ≥8 (7 IPLs) disease with tumor involving at least 20% of the tissue in the biopsy core. One patient had two discrete IPLs, each with GS 6 disease. Table 1 outlines the clinical characteristics of each patient and IPL included in the analysis. All patients were male and one patient was African American. Table 2 outlines the radiographic characteristics of each IPL included in the analysis. All low risk IPLs had a GS of 6. For high risk IPLs, GS ranged from 8 to 10. Among the specimens analyzed, PSA ranged from 2.95 to 15.2. Only one patient had palpable disease on DRE. All but three patients had previous extended-sextant prostate biopsies with median of 1 biopsy prior to MRI/TRUS fusion biopsy. Seven (58%) IPLs were located in the peripheral zone (PZ). The median radiographic prostate volume was 40.74 mL. The median greatest dimension of each IPL was 1.85 cm, and the median volume was 1.47 mL.

**Table 1:** Clinical characteristics of each patient and intraprostatic lesion (IPL)

Patient	Gleason score	Clinical stage	PSA prior to biopsy	Previous biopsies
1	3 + 3 = 6	T1c	4.1	0
2	3 + 3 = 6	T2a	2.95	1
3a	3 + 3 = 6	T1c	5.17	1
3b	3 + 3 = 6	T1c	5.17	1
4	3 + 3 = 6	T1c	6	1
5	3 + 3 = 6	T1c	4.5	0
6	3 + 3 = 6	T1c	5	4
7	4 + 4 = 8	T1c	9	2
8	4 + 4 = 8	T1c	5.1	1
9	4 + 5 = 9	T1c	15.2	4
10	4 + 5 = 9	T1c	9.02	2
11	5 + 5 = 10	T1c	6.19	0

**Table 2:** Radiographic characteristics of each intraprostatic lesion (IPL)

Patient	Gleason score	Lesion location	Lesion diameter (cm)	Lesion volume (mL)	PI-RADS v2 score	DCE
1	3 + 3 = 6	PZ	1.4	0.55	3	+
2	3 + 3 = 6	CG	2.3	2.20	4	+
3a	3 + 3 = 6	CG	2.4	1.42	4	-
3b	3 + 3 = 6	PZ	2.2	1.76	4	-
4	3 + 3 = 6	PZ	1.6	1.10	4	++
5	3 + 3 = 6	PZ	1.5	0.62	3	+
6	3 + 3 = 6	CG	2	1.56	5	++
7	4 + 4 = 8	PZ	1.8	1.74	5	++
8	4 + 4 = 8	PZ	1.3	2.08	5	++
9	4 + 5 = 9	CG	1.9	1.39	4	++
10	4 + 5 = 9	CG	1.8	1.21	5	++
11	5 + 5 = 10	PZ	2.8	1.52	5	++

PZ (peripheral zone). CG (central gland). DCE (dynamic contrast enhancement). – (none). + (moderate DCE). ++ (intense DCE).

#### Nanostring gene expression and pancancer pathways analysis

**Figure 1:**
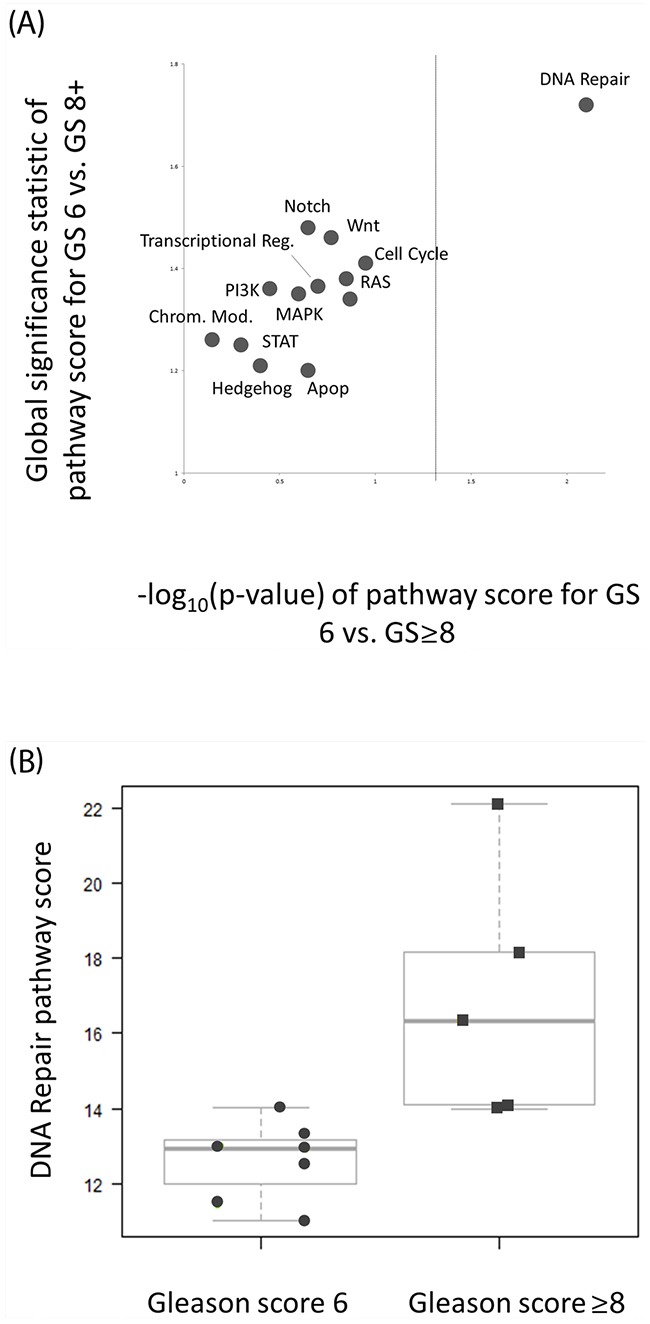
DNA Repair pathway deregulation is significantly associated with GS ≥8 intra-prostatic lesions **(A)** Pathway significance plot for pathway deregulation comparing GS 6 and GS ≥8 lesions. The global significance statistic (y-axis) represents the extent of differential deregulation of the DNA Repair pathway between GS 6 and GS ≥8 lesions. The p-value (x-axis) represents t-test comparison of pathway scores between groups (dashed line represents p = 0.05). Both tests are in agreement to the extent of DNA repair deregulation between groups. **(B)** Quantitative DNA Repair pathway scores were significantly higher for high risk (GS ≥8) versus low risk (GS 6) lesions. The magnitude of the pathway score represents the magnitude of pathway deregulation.

Gene expression analysis was performed using the Nanostring platform. Two (14%) samples had inadequate RNA to perform gene expression analysis, and both were GS ≥8. Gene expression and pathway analysis was performed on 12 specimens (7 GS 6 and 5 GS ≥8). Canonical cancer pathway-based gene expression analysis demonstrated significantly higher deregulation of the DNA Repair pathway in GS ≥8 IPLs compared to GS 6 (global significance statistic 1.7, p < 0.01, Figure 1). DNA repair deregulation was also strongly correlated with pre-biopsy PSA level (r = 0.81, p < 0.001)), and IPLs with PSA ≥ 10 had significantly higher DNA Repair deregulation than those with PSA < 10. IPLs with intense DCE had significantly higher DNA repair deregulation than those with no-to-moderate DCE (p = 0.03). IPLs with a PI-RADS score of 5 had significantly more deregulation of TGFβ, STAT, RAS, Apoptosis, and Cell Cycle pathways (p < 0.05, unadjusted for multiple testing) than IPLs with PI-RADS < 5.

**Figure 2:**
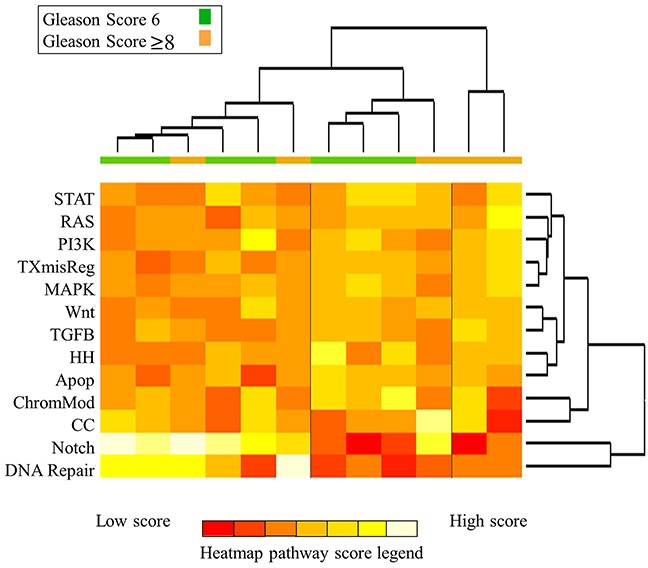
Heatmap of pathway scores for all intra-prostatic lesions Samples are colored according to Gleason score (green, GS 6; orange, GS ≥8). On the heatmap, red indicates low pathway score and yellow/white indicates high pathway score. Samples cluster into three clusters, largely based upon differential deregulation of (1) DNA Repair and Notch, (2) Chromatin Modification and Cell Cycle, and (3) all remaining pathways. GS 6 IPLs demonstrate heterogeneous levels of DNA Repair deregulation while GS ≥8 IPLs mainly show intermediate to high deregulation.

**Figure 3:**
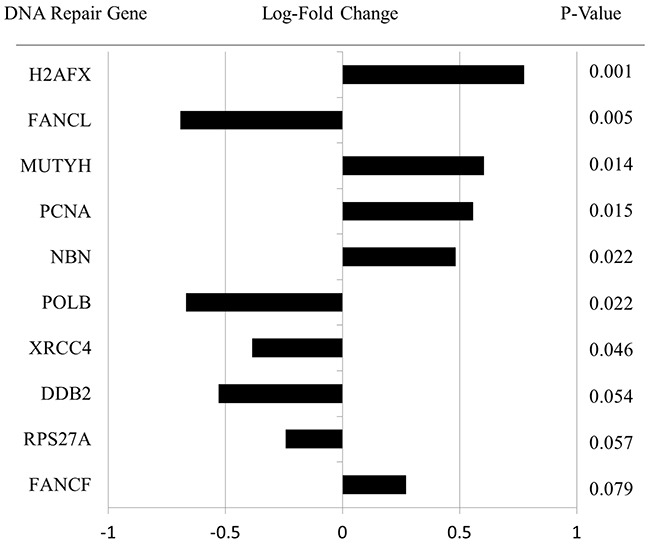
Specific DNA Repair pathway gene expression data for all lesions These genes represent the 10 most differentially expressed DNA Repair genes for GS ≥8 versus GS 6 with a positive value corresponding to increased expression of that gene in GS ≥8 versus GS 6 IPLs. The magnitude and direction of log-fold change in gene expression is shown along with p-value of the significance of difference between GS 6 and GS ≥8 IPLs.

On advanced pathway analysis as shown in Figure 2, IPLs generally grouped into three distinct clusters based on pathway deregulation. IPLs primarily grouped according to deregulation of the DNA Repair and Notch signaling pathways, followed by Cell Cycle and Chromatin Modification pathways. Pathway-based clustering showed heterogeneity between GS 6 and GS ≥8 lesions, but GS ≥8 lesions generally showed intermediate to high levels of DNA Repair and Notch deregulation. Supplementary Figure 1 shows the pathway heatmap in the context of clinical and radiographic characteristics. Figure 3 shows the 10 DNA Repair genes with the greatest differential expression between GS 6 and GS ≥8, and Supplementary Figure 2 shows the magnitude and significance of differential gene expression for all DNA Repair genes in a volcano plot. DNA Repair deregulation resulted from a variety of changes in gene expression with no dominant driver gene. Supplementary Table 1 lists all genes included in the analysis and the magnitude and significance of their differential expression.

## DISCUSSION

This study demonstrates the feasibility of advanced gene expression analysis using tissue obtained from targeted MRI/TRUS fusion-guided prostate biopsies. We found demonstrable differences in DNA repair defects between GS ≥8 and GS 6 IPLs, between IPLs with high and low PSA, and between IPLs with intense and no-to-moderate DCE. These findings correspond with other major studies describing a sizeable proportion of primary prostate cancers (∼20%) and castration-resistant prostate cancers (∼30%) driven by DNA repair defects [14, 15]. However, specific gene mutations resulting in DNA repair defects in prostate cancer are diverse, supporting the pathway approach to analysis utilized in this study. Both somatic and germline DNA repair defects appear to be associated with clinically significant disease [15, 17]. Importantly, DNA repair defects are targets of many emerging therapies, and DNA repair profiles of prostatectomy specimens are prognostic in men with high risk disease [15, 18]. Our findings correspond with larger studies in showing an association between DNA Repair defects and both Gleason score and PSA. We also found significant differences in expression of a variety of individual DNA repair genes between groups supporting the idea that pathway-level defects in prostate cancer are less commonly the result of individual driver genes.

Information about tumor biology at the time of biopsy would be most beneficial in guiding management, and the techniques used in this study represent a next-generation approach to prostate cancer diagnosis and risk stratification [19]. Identification of underlying molecular features of dominant IPLs at the time of diagnosis instead of after prostatectomy or at time of recurrence has important therapeutic implications. Defective DNA repair in metastatic, castration resistant prostate cancer is an appealing target of poly(ADP-ribose) polymerase (PARP) inhibitors and platinum chemotherapy [15]. Furthermore, androgen receptor signaling can increase transcription of several DNA repair genes [20]. Many of these AR-target DNA repair genes with increased expression in prostate cancer also had higher expression levels in the high-risk IPLs in this study. PARP-1, in addition to its role in DNA repair, is also thought to be an important promoter of androgen receptor activity and cell proliferation. In the context of these and other findings, subsets of high-risk prostate cancers with positive DNA repair deregulation may potentially benefit from the combination of androgen deprivation, which is currently standard of care, with PARP inhibitors to enhance tumor control. Cell cycle progression scores are also associated with risk of recurrence of prostate cancer, but appear to be less correlated with traditional clinical characteristics [21]. In this study, a subgroup of IPLs clustered according to cell cycle deregulation, and PIRADS score was also associated with the extent of cell cycle deregulation. The lack of a strong difference in cell cycle deregulation may result from the small sample size and reliance of traditional clinical features for comparison. While these findings echo those of larger studies, demonstrating the feasibility of a precision medicine approach to prostate cancer in routine clinical practice is meaningful. The platform used in this study also demonstrates the feasibility of using a decentralized testing process that can be performed on small quantity FFPE samples with short turnaround time.

High risk radiographic features, PI-RADS score and intensity of DCE, also strongly correlated with pathway deregulation. When interpreting these findings, it is important to note that high-risk imaging features are known to be associated with high-risk pathological features. One benefit of prostate MRI is increased detection of high risk lesions and decreased detection of low risk lesions. An important future goal of prostate MRI is to have imaging that highly correlates with pathological findings to avoid the need for biopsy in the setting of surveillance [23]. One purpose of this study was to demonstrate the feasibility of associating high-risk imaging features (known to correlate with pathological features) with biological changes of discrete IPLs. Of note, only the subset of IPLs with intense DCE demonstrated more deregulation. There was no difference in pathway scores on comparison of any DCE versus none. Development of radiographic biomarkers could significantly benefit the growing proportion of men who pursue active surveillance or radiotherapy in addition to better informing preoperative management. The techniques in this study could also be extrapolated to the recurrent or metastatic setting to better understand the biology of prostate cancer progression and recurrence.

This study is limited by its retrospective, exploratory nature and the small sample size. With a small sample size, the genomic heterogeneity of prostate cancer and potential inclusion of outlier mutations could potentially influence the results. However, the findings of DNA repair deregulation are consistent with findings of larger studies. Also, we focused on pathway, as opposed to specific gene, analysis to partially account for expected genomic heterogeneity. Small sample size also limits the interpretation of associations among clinical, radiographic, and molecular findings with multivariate analysis. In particular, many of the clinical and radiographic findings are known to be associated, such as high Gleason grade with PIRADS score and contrast enhancement or elevated PSA with high Gleason grade. The small sample size precludes more extensive statistical analysis that would be needed to demonstrate independent associations among various clinical and radiographic features and the molecular findings.

In conclusion, this study demonstrates the feasibility of obtaining detailed information about the molecular biology of discrete IPLs using mpMRI, MRI/TRUS fusion biopsy, and targeted gene expression and pathway analysis. Using this technique, we found significantly higher deregulation of DNA repair in high-grade lesions. We also found strong clinical and radiographic correlates of these underlying molecular changes. These techniques can be used in larger clinical trials to personalize definitive therapy and improve outcomes for clinically significant prostate cancer.

## MATERIALS AND METHODS

### Study population

Investigation has been conducted in accordance with the ethical standards and according to the Declaration of Helsinki and according to national and international guidelines and has been approved by the authors’ institutional review board. Patients who had targeted MRI/TRUS fusion-guided biopsy of IPLs with pathology demonstrating either low-risk (GS 6 and PSA < 10) or high-risk (GS ≥ 8 with any PSA) prostate cancer between December 2014 and December 2015 were identified. Because the purpose of this study was to determine the feasibility of detecting changes in cancer biology in a small sample size, only low-risk and high-risk histologies were compared in order to increase the likelihood of detecting a difference. Furthermore, Gleason 7 disease is known to be heterogeneous in both clinical and biological behavior. Multi-parametric prostate MRIs were reviewed by a multidisciplinary team of urologic oncologists and MRI radiologists to determine the risk of a lesion. IPLs with suspicious T2-weighted, diffusion-weighted, and dynamic contrast enhanced MR imaging were identified and a PI-RADS v2 score (MRI measure of prostate cancer suspicion, range 1-5) was assigned [7]. The extent of contrast enhancement was graded 0 for none, 1 for moderate degree of early enhancement with or without washout kinetics, and 2 for intense early enhancement with early washout characteristics. Grading of DCE was qualitative and performed by the team of prostate radiologists and urologists. Generally, all lesions felt to be at high risk of malignancy were biopsied using MRI/TRUS fusion biopsy. PSA at the time of biopsy and intra-prostatic location of each IPL were recorded.

### Gene expression profiling

All cases were reviewed by a single, fellowship trained genitourinary pathologist. Tumor foci from the MR/TRUS fusion biopsy specimens were identified and given a Gleason score. The pathologist determined if adequate tissue was available for molecular analysis. In cases where patients also had standard extended-sextant biopsy or subsequent radical prostatectomy, those tissue samples were not included in the analysis in order to demonstrate the feasibility of this technique using targeted biopsy samples only. RNA was isolated from FFPE prostate tumor tissue as previously described [22]. Briefly, hematoxylin and eosin (H&E) stained slides were examined and the areas of tumor suitable for analysis were identified. The corresponding areas on unstained slides were microdissected and digested with Proteinase K and DNase I as instructed in the High Pure FFPET RNA Isolation Kit (Roche Diagnostics, IN); the number of slides required depended on the surface area of the identified region with a section thickness of 10uM. A total of 100ng of isolated RNA was used for each analysis whenever possible. RNA was incubated overnight with the Reporter and Capture CodeSet probes specific to 770 cancer-related genes in the premade PanCancer Pathways panel (Nanostring Technologies, Seattle, WA; performed at UAB Nanostring Core Facility (http://www.uab.edu/medicine/radonc/en/nanostring)). The Reporter probe carries the fluorescent signal specific to each gene, and the Capture probe allows the complex to be immobilized on a cartridge for data collection. After hybridization, the samples were loaded into the nCounter Prep Station where excess probes were removed and the samples were aligned in order to be read properly in the nCounter Digital Analyzer.

### Cancer pathway and statistical analysis

The raw data was analyzed using a cancer pathway-based gene expression profiling platform. We then performed an advanced analysis of gene expression and cancer pathway signature data using the nSolver advanced analysis package. The primary outcome was differences in pathway deregulation scores for GS 6 and GS ≥8 IPLs calculated by the advanced gene expression analysis software using the t-test. Secondary outcomes were differences in pathway deregulation scores and correlation of scores with clinical and radiographic characteristics. Pathway deregulation scores were correlated with pre-biopsy serum PSA and PI-RADS score assigned to the specific IPL based upon pre-biopsy mpMRI. Pathway deregulation scores were also compared between IPLs with PI-RADS score 3-4 and 5, no/moderate and intense DCE, and central gland versus peripheral zone anatomic location in the prostate using the t-test and the built-in statistical software of the gene expression analysis platform.

## SUPPLEMENTARY MATERIALS FIGURES AND TABLE




