# Supplementary material for: DNA repair deregulation in discrete prostate cancer lesions identified on multi-parametric MRI and targeted by MRI/ultrasound fusion-guided biopsy
Source: Oncotarget. 2017 Jul 10;8(40):68038–46. doi: 10.18632/oncotarget.19145 (PMC5620234; doi:10.18632/oncotarget.19145)
Supplement: Supplementary file 1 [file oncotarget-08-68038-s001.pdf]

DNA repair deregulation in discrete prostate cancer lesions identified on multi-parametric MRI and targeted by MRI/ ultrasound fusion-guided biopsy

SUPPLEMENTARY MATERIALS

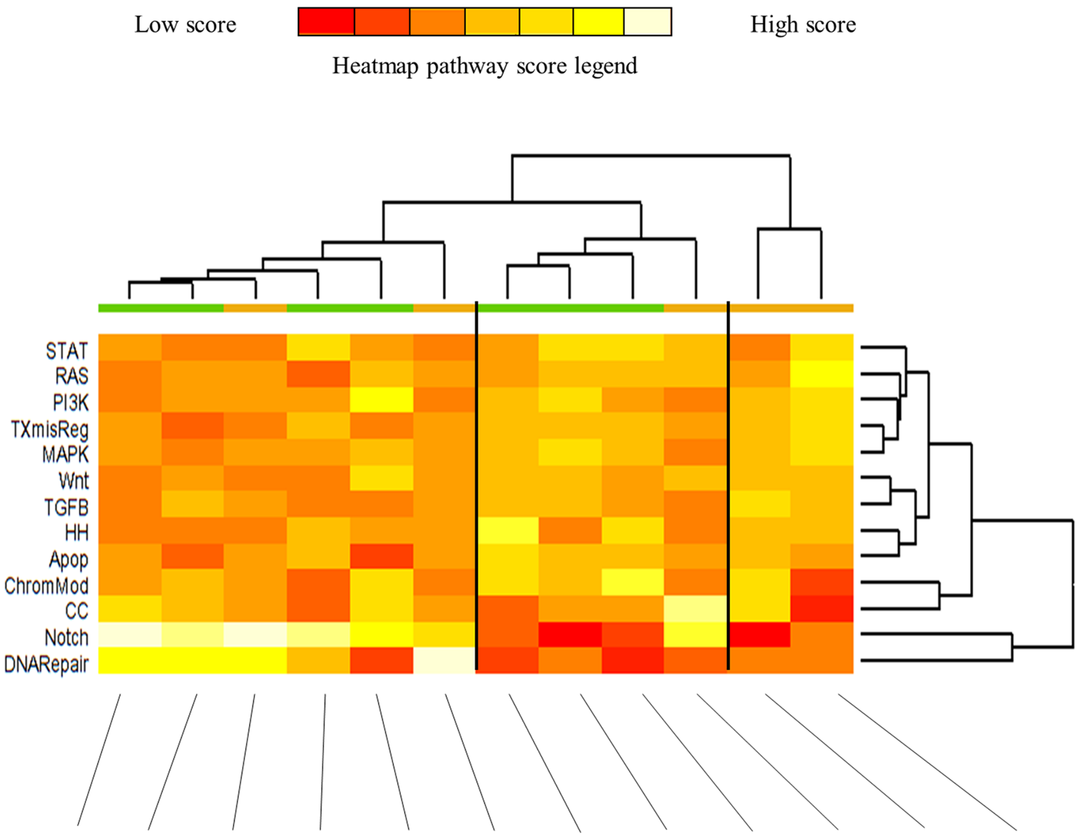

Patient	3B	4	7	3A	5	9	1	2	6	11	10	8
GS	6	6	8	6	6	9	6	6	6	10	9	8
PSA	5.17	6.0	5.1	5.17	4.5	15.2	4.1	2.95	5.0	6.19	9.02	5.1
PIRADS	4	4	5	4	3	4	3	4	5	5	5	5
DCE	-	++	++	-	+	++	++	+	++	++	++	++

Supplementary Figure 1: Heat map of pathway scores or all intra-prostatic lesions and associated clinical and radiographic characteristics. Samples clustered into three groups based on molecular characteristics, but each group had relatively heterogeneous clinical characteristics. Future work will aim to determine how traditionally high-risk lesions with low-risk molecular features or low-risk lesions with high-risk molecular features behave clinically.

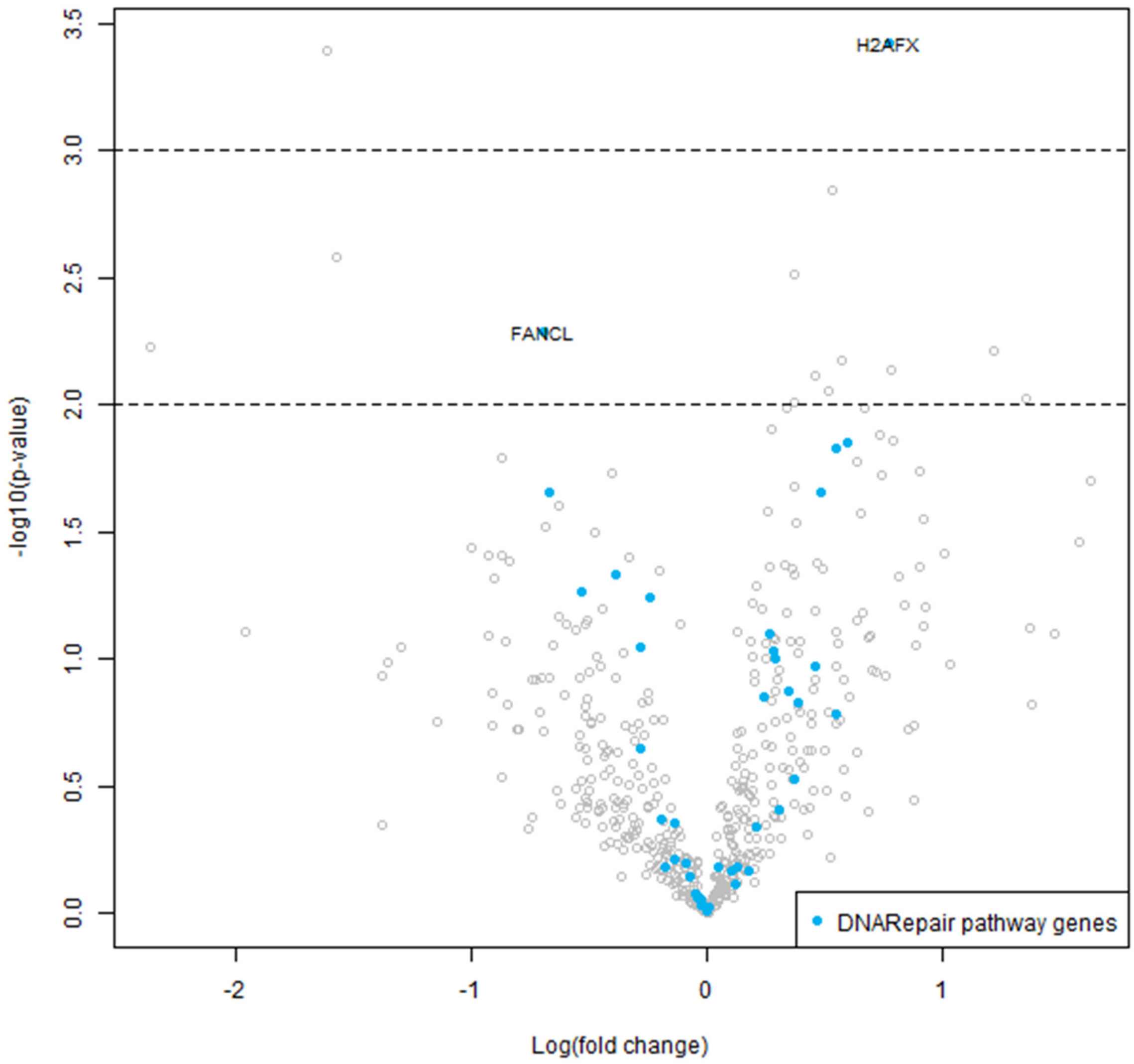

Supplementary Figure 2: Volcano plot showing differential gene expression for GS ≥8 lesions compared GS 6 lesions. Individual DNA Repair genes are highlighted in blue. The log-fold change in gene expression is plotted against the $-\log_{10}$ of the p-value from the comparison of gene expression between groups. The distance right or left of a gene on the graph corresponds to the magnitude of increased or decreased expression. The height of a gene on the graph corresponds to a lower p-value.

Supplementary Table 1: All genes included in the gene expression analysis organized by pathway.

See Supplementary File 1
