# Supplementary material for: DNA repair deregulation in discrete prostate cancer lesions identified on multi-parametric MRI and targeted by MRI/ultrasound fusion-guided biopsy
Source: Oncotarget. 2017 Jul 10;8(40):68038–46. doi: 10.18632/oncotarget.19145 (PMC5620234; doi:10.18632/oncotarget.19145)
Supplement: Supplementary file 2 [file oncotarget-08-68038-s002.docx]

**Supplementary Table 1: All genes included in the gene expression analysis organized by pathway.**

| **Gene** | **Pathways** | **Log fold change** | **P-value** |
| --- | --- | --- | --- |
| IRAK2 | Apop | -0.35099 | 0.094714 |
| PRKAR2A | Apop | 0.194171 | 0.327626 |
| TNFSF10 | Apop | -0.54036 | 0.118166 |
| ENDOG | Apop | 0.146627 | 0.614197 |
| IRAK3 | Apop | -0.34498 | 0.184141 |
| MYD88 | Apop | -0.23148 | 0.268141 |
| CASP8 | Apop | -0.09628 | 0.610267 |
| TNFRSF10A | Apop | -0.15196 | 0.696836 |
| BID | Apop | -0.09368 | 0.654339 |
| IL1RAP | Apop | 0.057691 | 0.831235 |
| TNFRSF10B | Apop | -0.30681 | 0.451512 |
| NFKBIA | Apop | -0.00555 | 0.96264 |
| BAX | Apop | 0.211115 | 0.051772 |
| PRKAR1B | Apop | 0.179667 | 0.586022 |
| PRKAR2B | Apop | 0.009564 | 0.972897 |
| CASP7 | Apop | -0.3769 | 0.301969 |
| CAPN2 | Apop | -0.13712 | 0.488598 |
| CDC14B | CC | 0.507603 | 0.330175 |
| CDKN1C | CC | -0.28381 | 0.284581 |
| SMC1A | CC | 0.160243 | 0.319489 |
| ATR | CC | -0.0252 | 0.885429 |
| RB1 | CC | 0.027719 | 0.867122 |
| CCNA2 | CC | 0.290435 | 0.177052 |
| E2F1 | CC | -0.16222 | 0.742292 |
| CDC6 | CC | 0.760861 | 0.115772 |
| MCM2 | CC | 0.050532 | 0.874679 |
| WEE1 | CC | 0.092673 | 0.693398 |
| SKP2 | CC | -0.16613 | 0.494081 |
| CDC14A | CC | -0.06071 | 0.917614 |
| RAD21 | CC | 0.462664 | 0.007635 |
| TTK | CC | 0.933298 | 0.062529 |
| CHEK2 | CC | 0.354708 | 0.203721 |
| SFN | CC | -0.73846 | 0.418452 |
| MCM7 | CC | 0.153966 | 0.300974 |
| SMC3 | CC | 0.19001 | 0.085492 |
| PTTG2 | CC | 0.504901 | 0.230715 |
| MCM5 | CC | 0.081118 | 0.633301 |
| MCM4 | CC | 0.566513 | 0.173004 |
| STAG2 | CC | 0.202356 | 0.121594 |
| CCNB1 | CC | 0.432015 | 0.227846 |
| HDAC6 | ChromMod | 0.099886 | 0.498278 |
| HELLS | ChromMod | 0.55543 | 0.106354 |
| HDAC4 | ChromMod | -0.01175 | 0.927231 |
| NASP | ChromMod | 0.140152 | 0.591972 |
| HDAC11 | ChromMod | 0.127591 | 0.577004 |
| ARID1A | ChromMod | 0.25564 | 0.087028 |
| WHSC1L1 | ChromMod | -0.05358 | 0.782722 |
| NSD1 | ChromMod | 0.053343 | 0.583422 |
| HMGA1 | ChromMod | 0.795973 | 0.013837 |
| PPARGC1A | ChromMod | 0.550359 | 0.178323 |
| HDAC10 | ChromMod | -0.14952 | 0.633559 |
| SUV39H2 | ChromMod | 0.018128 | 0.947682 |
| BNIP3 | ChromMod | -0.17093 | 0.660703 |
| HDAC5 | ChromMod | -0.08164 | 0.601544 |
| RPS6KA5 | ChromMod, MAPK | 0.279231 | 0.14523 |
| POLD4 | DNARepair | 0.131036 | 0.660247 |
| ALKBH3 | DNARepair | -0.04321 | 0.834315 |
| UBB | DNARepair | 0.287616 | 0.092954 |
| RPS27A | DNARepair | -0.24056 | 0.056851 |
| RAD50 | DNARepair | 0.105164 | 0.683358 |
| UBE2T | DNARepair | 0.312757 | 0.390275 |
| LIG4 | DNARepair | -0.06858 | 0.712902 |
| ERCC6 | DNARepair | -0.13672 | 0.442901 |
| RAD52 | DNARepair | 0.004214 | 0.976719 |
| H2AFX | DNARepair | 0.774888 | 0.000374 |
| FEN1 | DNARepair | 0.216962 | 0.456572 |
| MGMT | DNARepair | 0.012848 | 0.944418 |
| FANCE | DNARepair | 0.553334 | 0.163381 |
| ALKBH2 | DNARepair | -0.13577 | 0.616203 |
| FANCL | DNARepair | -0.69106 | 0.005128 |
| MNAT1 | DNARepair | -0.0204 | 0.924743 |
| NTHL1 | DNARepair | -0.18887 | 0.426148 |
| XPA | DNARepair | 0.055272 | 0.660328 |
| NBN | DNARepair | 0.483365 | 0.022068 |
| RFC3 | DNARepair | 0.182374 | 0.677208 |
| ERCC2 | DNARepair | -0.08205 | 0.632788 |
| FANCF | DNARepair | 0.270717 | 0.079193 |
| POLR2D | DNARepair | -0.01838 | 0.889655 |
| GTF2H3 | DNARepair | -0.27788 | 0.089626 |
| POLR2J | DNARepair | 0.45943 | 0.107473 |
| BRIP1 | DNARepair | -0.17022 | 0.655878 |
| POLR2H | DNARepair | 0.243449 | 0.140804 |
| DDB2 | DNARepair | -0.52837 | 0.054156 |
| POLB | DNARepair | -0.66848 | 0.022207 |
| RFC4 | DNARepair | 0.371127 | 0.294595 |
| FANCC | DNARepair | -0.27871 | 0.225078 |
| POLE2 | DNARepair | 0.120949 | 0.773948 |
| MUTYH | DNARepair | 0.602962 | 0.013997 |
| XRCC4 | DNARepair | -0.38475 | 0.046323 |
| FANCG | DNARepair | -0.04563 | 0.832941 |
| MDC1 | DNARepair | -0.03304 | 0.863174 |
| PRKDC | DNARepair, CC | 0.349537 | 0.134666 |
| MAD2L2 | DNARepair, CC | 0.388024 | 0.148465 |
| PCNA | DNARepair, CC | 0.555662 | 0.014897 |
| BRCA1 | DNARepair, PI3K | 0.293343 | 0.099467 |
| ATM | TXmisReg, DNARepair, Apop, CC | -0.03246 | 0.874938 |
| SMO | HH | -0.14648 | 0.521324 |
| GLI1 | HH | -0.20168 | 0.645012 |
| GLI3 | HH | -0.23098 | 0.539612 |
| HHIP | HH | -1.35099 | 0.103219 |
| PTCH1 | HH | -0.17445 | 0.585296 |
| GAS1 | HH | 0.105247 | 0.779554 |
| ZIC2 | HH | 0.007333 | 0.995548 |
| BMP4 | HH, TGFB | -0.50289 | 0.247882 |
| HSPA6 | MAPK | 0.012547 | 0.973187 |
| MAPT | MAPK | 0.217628 | 0.607424 |
| CACNA1D | MAPK | -0.53135 | 0.302153 |
| HSPA1A | MAPK | 0.034123 | 0.933905 |
| CACNB2 | MAPK | 0.128467 | 0.756007 |
| CACNA2D2 | MAPK | 0.167033 | 0.68751 |
| MAP3K1 | MAPK | -0.11753 | 0.470382 |
| HSPB1 | MAPK | 0.783634 | 0.007264 |
| FLNA | MAPK | -0.49701 | 0.331511 |
| FLNC | MAPK | -0.37906 | 0.535455 |
| MAP2K6 | MAPK | -1.60561 | 0.000399 |
| CACNA2D1 | MAPK | -0.51046 | 0.354554 |
| FOS | MAPK | -0.45942 | 0.400002 |
| DUSP10 | MAPK | 0.339157 | 0.066078 |
| CACNB3 | MAPK | 0.705298 | 0.111118 |
| MAPK12 | MAPK | 0.202012 | 0.67077 |
| MAP2K4 | MAPK | 0.001079 | 0.994589 |
| BRAF | MAPK | 0.110338 | 0.476957 |
| ZAK | MAPK | -0.08548 | 0.817516 |
| CACNA1H | MAPK | 0.641365 | 0.232457 |
| HSPA2 | MAPK | 0.450172 | 0.178687 |
| CACNA1C | MAPK | -0.29915 | 0.487976 |
| DAXX | MAPK | 0.130702 | 0.317705 |
| MECOM | MAPK | 0.364247 | 0.298763 |
| MAP3K8 | MAPK | -0.40434 | 0.272334 |
| MAPK8IP2 | MAPK | 1.581782 | 0.034884 |
| STMN1 | MAPK | 0.285101 | 0.414124 |
| DUSP5 | MAPK | -0.54103 | 0.381941 |
| RPS6KA6 | MAPK | 0.028055 | 0.879241 |
| CACNG4 | MAPK | -0.55298 | 0.419497 |
| MAP3K13 | MAPK | -0.62859 | 0.024708 |
| MAP3K5 | MAPK | 0.440254 | 0.387438 |
| NTRK2 | MAPK | -0.07169 | 0.906852 |
| CACNA2D3 | MAPK | -0.161 | 0.634371 |
| DUSP8 | MAPK | 0.452408 | 0.131534 |
| CASP3 | MAPK, Apop | 0.152895 | 0.426601 |
| IL1R1 | MAPK, Apop | -0.00603 | 0.984633 |
| MAP3K14 | MAPK, Apop | 0.001524 | 0.994227 |
| FAS | MAPK, Apop | -0.50953 | 0.072747 |
| GADD45G | MAPK, CC | 0.450867 | 0.16395 |
| GADD45B | MAPK, CC | -0.00596 | 0.982147 |
| GADD45A | MAPK, CC | -0.38449 | 0.119263 |
| CDC25B | MAPK, CC | -0.03797 | 0.873928 |
| NR4A1 | MAPK, PI3K | -0.34885 | 0.381804 |
| NRAS | MAPK, PI3K, RAS | -0.07778 | 0.610226 |
| HRAS | MAPK, PI3K, RAS | 0.574134 | 0.006648 |
| KRAS | MAPK, PI3K, RAS | 0.492685 | 0.043881 |
| GNG12 | MAPK, PI3K, RAS | -0.24452 | 0.146476 |
| MAP2K1 | MAPK, PI3K, RAS | 0.058477 | 0.833132 |
| PDGFRA | MAPK, PI3K, RAS | -0.38913 | 0.352701 |
| RAF1 | MAPK, PI3K, RAS | 0.375288 | 0.003039 |
| FGF2 | MAPK, PI3K, RAS | -0.9221 | 0.039241 |
| FGFR3 | MAPK, PI3K, RAS | 0.233891 | 0.587801 |
| FGF7 | MAPK, PI3K, RAS | -0.44236 | 0.217392 |
| FGF13 | MAPK, PI3K, RAS | -0.12642 | 0.619801 |
| EGFR | MAPK, PI3K, RAS | 0.055566 | 0.82845 |
| FGFR2 | MAPK, PI3K, RAS | 0.37404 | 0.372018 |
| FGFR1 | MAPK, PI3K, RAS | -0.28425 | 0.442052 |
| EGF | MAPK, PI3K, RAS | -0.9117 | 0.18215 |
| PDGFRB | MAPK, PI3K, RAS | 0.065782 | 0.775832 |
| MAP2K2 | MAPK, PI3K, RAS | 0.194739 | 0.059813 |
| IKBKG | MAPK, PI3K, RAS, Apop | 0.181693 | 0.339845 |
| CHUK | MAPK, PI3K, RAS, Apop | 0.128295 | 0.224555 |
| IKBKB | MAPK, PI3K, RAS, Apop | -0.55646 | 0.076684 |
| PLA2G4A | MAPK, RAS | -0.69665 | 0.117855 |
| PLA2G4C | MAPK, RAS | -0.51127 | 0.44492 |
| RRAS2 | MAPK, RAS | 0.059201 | 0.764603 |
| RASGRP2 | MAPK, RAS | 0.113556 | 0.780809 |
| PLA2G4F | MAPK, RAS | -0.68956 | 0.191352 |
| NF1 | MAPK, RAS | -0.21957 | 0.305771 |
| SOS2 | MAPK, STAT, PI3K, RAS | 0.00394 | 0.988849 |
| SOS1 | MAPK, STAT, PI3K, RAS | -0.06057 | 0.636134 |
| GRB2 | MAPK, STAT, PI3K, RAS | -0.12165 | 0.584925 |
| AKT2 | MAPK, STAT, PI3K, RAS, Apop | -0.03592 | 0.789861 |
| AKT1 | MAPK, STAT, PI3K, RAS, Apop | 0.15065 | 0.193333 |
| AKT3 | MAPK, STAT, PI3K, RAS, Apop | 0.069428 | 0.828429 |
| KAT2B | Notch | -0.18077 | 0.437225 |
| MFNG | Notch | -0.13503 | 0.761313 |
| JAG1 | Notch | 0.884736 | 0.182557 |
| NOTCH3 | Notch | 0.69631 | 0.080339 |
| JAG2 | Notch | 0.580514 | 0.120161 |
| DTX4 | Notch | -0.05835 | 0.862145 |
| NOTCH1 | Notch | 0.517136 | 0.161147 |
| DTX1 | Notch | 0.463688 | 0.119795 |
| DLL1 | Notch | -0.05383 | 0.8652 |
| NOTCH2 | Notch | 0.33445 | 0.042241 |
| APH1B | Notch | -0.14377 | 0.46838 |
| DLL4 | Notch | 0.639361 | 0.016798 |
| MAML2 | Notch | -0.1164 | 0.740346 |
| LFNG | Notch | -0.9111 | 0.136708 |
| NUMBL | Notch | -0.46434 | 0.098364 |
| DTX3 | Notch | -0.10005 | 0.848584 |
| HES1 | Notch | -0.44916 | 0.370935 |
| HDAC2 | Notch, ChromMod, TXmisReg, CC | 0.306682 | 0.109658 |
| HDAC1 | Notch, ChromMod, TXmisReg, CC | -0.17805 | 0.298177 |
| EP300 | Notch, Wnt, TGFB, STAT, CC | 0.130066 | 0.07848 |
| ITGB8 | PI3K | 0.109979 | 0.809149 |
| PRKAA2 | PI3K | -0.86958 | 0.016272 |
| ITGA2 | PI3K | -0.43821 | 0.063534 |
| COL4A6 | PI3K | -0.43315 | 0.241062 |
| ITGA7 | PI3K | -0.02405 | 0.960721 |
| TLR2 | PI3K | 0.160374 | 0.664873 |
| HSP90B1 | PI3K | -0.26652 | 0.198251 |
| PPP2R2C | PI3K | 1.356431 | 0.009444 |
| COL5A1 | PI3K | -0.12483 | 0.75935 |
| COMP | PI3K | 1.034726 | 0.105252 |
| CREB3L1 | PI3K | -0.51516 | 0.166457 |
| THBS4 | PI3K | 0.529268 | 0.600026 |
| ITGA6 | PI3K | -0.01071 | 0.972971 |
| THEM4 | PI3K | 0.035761 | 0.850566 |
| MYB | PI3K | -0.55255 | 0.324846 |
| DDIT4 | PI3K | -0.48755 | 0.17978 |
| SPP1 | PI3K | -0.89666 | 0.048272 |
| FN1 | PI3K | 0.045389 | 0.930478 |
| EIF4EBP1 | PI3K | -0.8666 | 0.038734 |
| COL4A5 | PI3K | -0.2194 | 0.174175 |
| SYK | PI3K | 0.399964 | 0.254412 |
| ITGA3 | PI3K | 0.072571 | 0.794018 |
| TLR4 | PI3K | -0.29113 | 0.473523 |
| IRS1 | PI3K | -0.34578 | 0.500713 |
| CREB5 | PI3K | 0.047925 | 0.899872 |
| ITGA8 | PI3K | -0.50443 | 0.366753 |
| CREB3L4 | PI3K | -0.00711 | 0.979577 |
| ITGB6 | PI3K | 0.266327 | 0.582371 |
| STK11 | PI3K | 0.060382 | 0.736379 |
| COL27A1 | PI3K | -0.61448 | 0.372637 |
| ITGB4 | PI3K | -0.35081 | 0.56435 |
| COL1A1 | PI3K | 0.818887 | 0.046963 |
| MTOR | PI3K | -0.64946 | 0.088271 |
| ITGB3 | PI3K | -0.20776 | 0.660767 |
| ITGA9 | PI3K | -0.18056 | 0.676967 |
| COL3A1 | PI3K | 0.097194 | 0.811679 |
| PTEN | PI3K | 0.167802 | 0.282878 |
| COL5A2 | PI3K | 0.318164 | 0.507962 |
| COL1A2 | PI3K | 0.081102 | 0.855204 |
| LAMB3 | PI3K | -0.86405 | 0.293022 |
| COL4A3 | PI3K | -0.99538 | 0.036736 |
| LAMA5 | PI3K | 0.176698 | 0.464229 |
| TSC1 | PI3K | 0.132061 | 0.195571 |
| BCL2 | PI3K, Apop | 0.663884 | 0.065712 |
| CASP9 | PI3K, Apop | -0.02526 | 0.94379 |
| CDK4 | PI3K, CC | 0.13777 | 0.407074 |
| CCNE1 | PI3K, CC | 0.352723 | 0.26987 |
| CDK2 | PI3K, CC | 0.207099 | 0.362348 |
| CDK6 | PI3K, CC | -0.35485 | 0.370731 |
| KITLG | PI3K, RAS | 0.608053 | 0.141077 |
| GNG4 | PI3K, RAS | -1.37953 | 0.11606 |
| EFNA5 | PI3K, RAS | 0.163346 | 0.662286 |
| ANGPT1 | PI3K, RAS | -0.70793 | 0.161749 |
| VEGFC | PI3K, RAS | 0.358419 | 0.08583 |
| GNG7 | PI3K, RAS | -0.05247 | 0.848396 |
| PDGFC | PI3K, RAS | -0.66844 | 0.118336 |
| HGF | PI3K, RAS | -0.24553 | 0.525834 |
| VEGFA | PI3K, RAS | 1.385357 | 0.149957 |
| EFNA3 | PI3K, RAS | 1.636014 | 0.019961 |
| EFNA1 | PI3K, RAS | 0.196797 | 0.235365 |
| KIT | PI3K, RAS | 0.639861 | 0.070155 |
| PGF | PI3K, RAS | -0.12943 | 0.861191 |
| PDGFD | PI3K, RAS | 0.593174 | 0.349575 |
| BAD | PI3K, RAS, Apop | 0.371809 | 0.009841 |
| RASA4 | RAS | -0.10163 | 0.804983 |
| LAT | RAS | 0.249426 | 0.450663 |
| FOXO4 | RAS | -0.21935 | 0.37203 |
| PLCE1 | RAS | 0.400469 | 0.160744 |
| SHC2 | RAS | 0.119165 | 0.71617 |
| SHC1 | RAS | 0.014102 | 0.917813 |
| PLA2G2A | RAS | -1.37207 | 0.453222 |
| TIAM1 | RAS | -0.2689 | 0.324591 |
| PLD1 | RAS | 0.690042 | 0.401239 |
| ETS2 | RAS | -0.33348 | 0.360227 |
| PLA1A | RAS | -2.35686 | 0.005926 |
| MLLT4 | RAS | 0.294597 | 0.132748 |
| PLCG2 | RAS | -0.44944 | 0.106377 |
| ABL1 | RAS, CC | -0.03754 | 0.619933 |
| IL11RA | STAT | 0.100635 | 0.667344 |
| TSLP | STAT | -0.29912 | 0.550699 |
| STAT1 | STAT | -0.54052 | 0.197953 |
| IL20RA | STAT | -0.68075 | 0.029921 |
| LIF | STAT | 0.068842 | 0.827456 |
| LIFR | STAT | 0.011042 | 0.951732 |
| SOCS3 | STAT | -0.19101 | 0.732848 |
| LEPR | STAT | -0.72497 | 0.120882 |
| SPRY2 | STAT | -0.17872 | 0.532127 |
| SOCS2 | STAT | 0.289275 | 0.36754 |
| SPRY4 | STAT | 0.925846 | 0.073794 |
| STAT3 | STAT | -0.05631 | 0.810099 |
| CNTFR | STAT | -0.40883 | 0.524888 |
| CLCF1 | STAT | -0.38887 | 0.436426 |
| CBL | STAT | 0.277927 | 0.221413 |
| CBLC | STAT | -0.84183 | 0.151245 |
| SPRY1 | STAT | -0.5107 | 0.226718 |
| PIM1 | STAT | 0.115272 | 0.70592 |
| IL15 | STAT | -0.25591 | 0.700377 |
| JAK1 | STAT, PI3K | -0.00962 | 0.945467 |
| JAK2 | STAT, PI3K | 0.072245 | 0.623076 |
| PRLR | STAT, PI3K | -1.57089 | 0.002628 |
| IL6R | STAT, PI3K | -0.37386 | 0.365459 |
| EPOR | STAT, PI3K | 0.16405 | 0.621499 |
| IL7R | STAT, PI3K | 0.113146 | 0.875516 |
| CSF3R | STAT, PI3K | 0.412829 | 0.391691 |
| JAK3 | STAT, PI3K | -0.20749 | 0.598861 |
| GHR | STAT, PI3K | -0.60499 | 0.137924 |
| PIK3R2 | STAT, PI3K, RAS, Apop | 0.038912 | 0.84356 |
| PIK3CA | STAT, PI3K, RAS, Apop | 0.154062 | 0.244626 |
| PIK3CG | STAT, PI3K, RAS, Apop | -0.223 | 0.57463 |
| PIK3R1 | STAT, PI3K, RAS, Apop | -0.24984 | 0.38498 |
| PIK3CD | STAT, PI3K, RAS, Apop | -0.24107 | 0.643395 |
| PIK3R5 | STAT, PI3K, RAS, Apop | -0.33089 | 0.511839 |
| PIK3R3 | STAT, PI3K, RAS, Apop | -0.18398 | 0.554411 |
| PIK3CB | STAT, PI3K, RAS, Apop | 0.675096 | 0.010312 |
| PTPN11 | STAT, RAS | 0.089096 | 0.421289 |
| INHBA | TGFB | 0.741925 | 0.018954 |
| BMP6 | TGFB | 1.476296 | 0.079351 |
| BMP7 | TGFB | -0.75261 | 0.466468 |
| FST | TGFB | -0.9248 | 0.080305 |
| ACVR1B | TGFB | -0.50564 | 0.070669 |
| BMP5 | TGFB | -0.35617 | 0.714566 |
| BMPR1B | TGFB | 0.366756 | 0.227102 |
| ID4 | TGFB | -0.43454 | 0.2843 |
| ACVR2A | TGFB | 0.188261 | 0.345001 |
| SMAD9 | TGFB | -0.35475 | 0.387633 |
| INHBB | TGFB | 0.586501 | 0.273007 |
| LTBP1 | TGFB | -0.44369 | 0.384999 |
| ID1 | TGFB | -0.04869 | 0.935285 |
| SMAD2 | TGFB, CC | -0.1848 | 0.174299 |
| TFDP1 | TGFB, CC | -0.03473 | 0.872369 |
| CDKN2B | TGFB, CC | 0.177837 | 0.504431 |
| E2F5 | TGFB, CC | 0.453666 | 0.328364 |
| TGFB1 | TGFB, MAPK, CC | -0.13181 | 0.707157 |
| TGFB3 | TGFB, MAPK, CC | 0.434021 | 0.489268 |
| TGFB2 | TGFB, MAPK, CC | -0.44715 | 0.170379 |
| MAPK3 | TGFB, MAPK, PI3K, RAS | 0.114938 | 0.5448 |
| MAPK1 | TGFB, MAPK, PI3K, RAS | 0.076933 | 0.633655 |
| PPP2CB | TGFB, PI3K | 0.024942 | 0.900591 |
| PPP2R1A | TGFB, PI3K | 0.090667 | 0.465062 |
| THBS1 | TGFB, PI3K | 0.087694 | 0.833696 |
| ETV1 | TXmisReg | -0.31386 | 0.473193 |
| NFKBIZ | TXmisReg | 0.558073 | 0.08622 |
| ZBTB16 | TXmisReg | 0.31712 | 0.419802 |
| MEN1 | TXmisReg | 0.340705 | 0.170746 |
| PPARG | TXmisReg | 0.854915 | 0.1884 |
| PBX1 | TXmisReg | -0.20819 | 0.345453 |
| KDM6A | TXmisReg | -0.20147 | 0.044599 |
| TMPRSS2 | TXmisReg | -0.31274 | 0.42153 |
| RUNX1T1 | TXmisReg | -0.16573 | 0.589835 |
| HHEX | TXmisReg | 0.009395 | 0.979482 |
| IGFBP3 | TXmisReg | 0.653196 | 0.026734 |
| EYA1 | TXmisReg | -0.626 | 0.068347 |
| NCOR1 | TXmisReg | 0.239131 | 0.184943 |
| BAIAP3 | TXmisReg | -0.02886 | 0.941328 |
| PML | TXmisReg | -0.11621 | 0.669886 |
| SIX1 | TXmisReg | 0.89121 | 0.087785 |
| HIST1H3H | TXmisReg | 0.845443 | 0.060973 |
| MLF1 | TXmisReg | -0.16614 | 0.674487 |
| ARNT2 | TXmisReg | -0.24726 | 0.135707 |
| HOXA10 | TXmisReg | 0.077111 | 0.765194 |
| SIN3A | TXmisReg | 0.141385 | 0.329217 |
| MLLT3 | TXmisReg | 0.059376 | 0.749997 |
| HIST1H3B | TXmisReg | 1.370605 | 0.076004 |
| TCF3 | TXmisReg | -0.2714 | 0.147126 |
| HOXA11 | TXmisReg | -0.30143 | 0.208735 |
| PLAT | TXmisReg | -0.41507 | 0.2302 |
| PLAU | TXmisReg | -0.50222 | 0.14272 |
| PBX3 | TXmisReg | 0.164962 | 0.425069 |
| HOXA9 | TXmisReg | 0.471633 | 0.041889 |
| GRIA3 | TXmisReg | -0.43354 | 0.54711 |
| H3F3A | TXmisReg | 0.206197 | 0.11464 |
| CD40 | TXmisReg | -0.51684 | 0.154097 |
| H3F3C | TXmisReg | 0.126666 | 0.639116 |
| TSPAN7 | TXmisReg | -0.09378 | 0.824584 |
| CEBPA | TXmisReg | 0.26705 | 0.267076 |
| WHSC1 | TXmisReg | 0.534498 | 0.00143 |
| FUT8 | TXmisReg | -0.33101 | 0.312584 |
| NUPR1 | TXmisReg | 0.212418 | 0.576734 |
| RUNX1 | TXmisReg | -0.38039 | 0.457623 |
| CDKN2C | TXmisReg, CC | 0.904685 | 0.018256 |
| CD14 | TXmisReg, MAPK | -0.0389 | 0.912089 |
| DUSP6 | TXmisReg, MAPK | -0.16511 | 0.563127 |
| DDIT3 | TXmisReg, MAPK | 0.329578 | 0.266919 |
| PDGFA | TXmisReg, MAPK, PI3K, RAS | 0.276621 | 0.310654 |
| RELA | TXmisReg, MAPK, PI3K, RAS, Apop | -0.00371 | 0.983692 |
| NFKB1 | TXmisReg, MAPK, PI3K, RAS, Apop | 0.015609 | 0.919296 |
| CDKN1A | TXmisReg, PI3K, CC | 0.71927 | 0.113353 |
| CDKN1B | TXmisReg, PI3K, CC | 0.400148 | 0.085886 |
| MDM2 | TXmisReg, PI3K, CC | 0.041328 | 0.502621 |
| NGFR | TXmisReg, PI3K, RAS | -0.63649 | 0.330447 |
| IGF1R | TXmisReg, PI3K, RAS | 0.446755 | 0.229627 |
| MET | TXmisReg, PI3K, RAS | -0.13971 | 0.772659 |
| IGF1 | TXmisReg, PI3K, RAS | 0.881675 | 0.356274 |
| FLT1 | TXmisReg, PI3K, RAS | 0.415897 | 0.26738 |
| IL2RB | TXmisReg, STAT, PI3K | -0.33566 | 0.505427 |
| BCL2L1 | TXmisReg, STAT, PI3K, RAS, Apop | -0.05301 | 0.76134 |
| SP1 | TXmisReg, TGFB | 0.008726 | 0.951318 |
| ID2 | TXmisReg, TGFB | -0.19595 | 0.505573 |
| TGFBR2 | TXmisReg, TGFB, MAPK | -0.31105 | 0.190478 |
| PLCB4 | Wnt | -0.59126 | 0.073516 |
| SFRP4 | Wnt | -0.43915 | 0.460376 |
| PLCB1 | Wnt | 0.028325 | 0.914083 |
| TBL1XR1 | Wnt | 0.296516 | 0.422523 |
| CTNNB1 | Wnt | 0.162332 | 0.390332 |
| SFRP2 | Wnt | -0.12182 | 0.856564 |
| FZD7 | Wnt | -0.06125 | 0.867535 |
| TCF7L1 | Wnt | -0.36778 | 0.425675 |
| MMP7 | Wnt | -1.95407 | 0.077741 |
| WIF1 | Wnt | -0.80718 | 0.188208 |
| CAMK2B | Wnt | 0.019557 | 0.959326 |
| SFRP1 | Wnt | -0.7976 | 0.190201 |
| APC | Wnt | -0.17628 | 0.582293 |
| FZD3 | Wnt | -0.47363 | 0.031533 |
| NKD1 | Wnt | -0.48697 | 0.177226 |
| GPC4 | Wnt | -0.73924 | 0.121316 |
| AXIN2 | Wnt | -0.01627 | 0.962044 |
| FZD8 | Wnt | -0.17865 | 0.673724 |
| AXIN1 | Wnt | 0.376334 | 0.020935 |
| LEF1 | Wnt | 0.017261 | 0.96809 |
| WNT4 | Wnt, HH | -0.45301 | 0.389428 |
| WNT5B | Wnt, HH | 0.137085 | 0.595429 |
| WNT7B | Wnt, HH | 0.20204 | 0.751873 |
| WNT6 | Wnt, HH | 1.012037 | 0.038721 |
| WNT5A | Wnt, HH | 1.221715 | 0.00611 |
| WNT2B | Wnt, HH | -0.25451 | 0.551378 |
| PRKACA | Wnt, HH, MAPK, RAS, Apop | -0.03892 | 0.631133 |
| PRKX | Wnt, HH, MAPK, RAS, Apop | -0.03746 | 0.896322 |
| PRKACB | Wnt, HH, MAPK, RAS, Apop | 0.270942 | 0.508632 |
| GSK3B | Wnt, HH, PI3K, CC | 0.008847 | 0.932873 |
| JUN | Wnt, MAPK | 0.117924 | 0.683614 |
| PPP3CA | Wnt, MAPK, Apop | 0.920105 | 0.027959 |
| PPP3R1 | Wnt, MAPK, Apop | 0.123783 | 0.26341 |
| PPP3CC | Wnt, MAPK, Apop | -0.29066 | 0.18073 |
| PPP3CB | Wnt, MAPK, Apop | 0.014059 | 0.956566 |
| RAC1 | Wnt, MAPK, PI3K, RAS | 0.050882 | 0.722123 |
| PRKCA | Wnt, MAPK, PI3K, RAS | -0.21272 | 0.674807 |
| RAC3 | Wnt, MAPK, RAS | 0.249766 | 0.218472 |
| MAPK9 | Wnt, MAPK, RAS | 0.389686 | 0.153957 |
| MAPK10 | Wnt, MAPK, RAS | 0.687458 | 0.082331 |
| RAC2 | Wnt, MAPK, RAS | -0.49244 | 0.294238 |
| PRKCB | Wnt, MAPK, RAS | -0.50104 | 0.386579 |
| MAPK8 | Wnt, MAPK, RAS | 0.239338 | 0.063743 |
| CCND3 | Wnt, STAT, PI3K, CC | -0.06233 | 0.833624 |
| CCND1 | Wnt, STAT, PI3K, CC | 0.73595 | 0.013212 |
| BAMBI | Wnt, TGFB | 0.908307 | 0.043371 |
| RBX1 | Wnt, TGFB, CC | 0.135026 | 0.391776 |
| CUL1 | Wnt, TGFB, CC | 0.273713 | 0.012511 |
| SMAD4 | Wnt, TGFB, CC | -0.32991 | 0.039624 |
| SKP1 | Wnt, TGFB, CC | 0.031384 | 0.885917 |
| SMAD3 | Wnt, TGFB, CC | -0.1474 | 0.548438 |
| RHOA | Wnt, TGFB, RAS | 0.298076 | 0.12126 |
| TP53 | Wnt, TXmisReg, MAPK, PI3K, Apop, CC | -0.1295 | 0.626975 |
| CCND2 | Wnt, TXmisReg, STAT, PI3K, CC | -0.42759 | 0.234146 |
| MYC | Wnt, TXmisReg, TGFB, MAPK, STAT, PI3K, CC | -0.38682 | 0.383537 |
| SETBP1 |  | -0.49472 | 0.112077 |
| SMARCA4 | | -0.03569 | 0.696429 |
| AR |  | 0.461324 | 0.064333 |
| TRAF7 |  | 0.366674 | 0.043775 |
| EZH2 |  | 0.54857 | 0.077799 |
| PBRM1 |  | 0.251239 | 0.099723 |
| TET2 |  | -0.11244 | 0.500957 |
| DNMT1 |  | -0.09198 | 0.722488 |
| SMARCB1 |  | 0.091218 | 0.410181 |
| RET |  | -1.14399 | 0.177147 |
| GNAQ |  | 0.272957 | 0.043114 |
| CIC |  | 0.10439 | 0.721124 |
| SOX9 |  | -1.29682 | 0.089391 |
| AMER1 |  | 0.117612 | 0.599477 |
| IDH1 |  | -0.83452 | 0.041404 |
| MLH1 |  | 0.200178 | 0.097407 |
| PRDM1 |  | 0.215425 | 0.505504 |
| GNA11 |  | 0.194506 | 0.289879 |
| MSH6 |  | 0.372746 | 0.046355 |
| CYLD |  | -0.00819 | 0.970074 |
| SETD2 |  | 0.108481 | 0.303775 |
| GNAS |  | 0.386161 | 0.029341 |
| SRSF2 |  | 0.124445 | 0.45566 |
| KMT2C |  | 0.11052 | 0.535541 |
| GATA2 |  | 0.392774 | 0.094707 |
| ERBB2 |  | 0.192229 | 0.593566 |
| U2AF1 |  | 0.063775 | 0.382947 |
| FBXW7 |  | 0.208411 | 0.426793 |
| ARID2 |  | 0.292785 | 0.084468 |
| SF3B1 |  | -0.24573 | 0.380745 |
| SPOP |  | 0.095496 | 0.471411 |
| ATRX |  | 0.102995 | 0.492263 |
| B2M |  | -0.53317 | 0.219845 |
| NF2 |  | 0.288716 | 0.081962 |
| KMT2D |  | 0.068851 | 0.381217 |
| BCOR |  | 0.523138 | 0.00886 |
| MED12 |  | -0.10946 | 0.072608 |
| RNF43 |  | 0.075633 | 0.850646 |
| ARID1B |  | -0.06374 | 0.68789 |
| KLF4 |  | -0.31213 | 0.257949 |
| TNFAIP3 |  | -0.85034 | 0.085127 |
| DNMT3A |  | 0.055836 | 0.759248 |
| FUBP1 |  | -0.40199 | 0.018574 |
| CDH1 |  | -0.37538 | 0.2342 |
| VHL |  | 0.344314 | 0.010241 |
| BAP1 |  | 0.265036 | 0.026125 |
| NFE2L2 |  | -0.05549 | 0.732603 |
| ASXL1 |  | 0.046063 | 0.719629 |
| IDH2 |  | -0.14799 | 0.382985 |
| MSH2 |  | 0.048709 | 0.794034 |
| PHF6 |  | 0.144836 | 0.326126 |
| KDM5C |  | 0.039333 | 0.712564 |

The log fold change in gene expression for GS ≥8 lesions compared to GS 6 lesions is shown along with the p-value for the comparison of means between groups using the t-test.
